# Notes on the Treatment of Acute Tonsillitis in Children

**Published:** 1888-05

**Authors:** Frank Hamilton Potter

**Affiliations:** Lecturer on Laryngology, Medical Department, Niagara University; 273 Franklin street


### *NOTES ON THE TREATMENT OF ACUTE TONSILLITIS IN CHILDREN.**

By FRANK HAMILTON POTTER, M. D.

Lecturer on Laryngology, Medical Department, Niagara University.

When an inflammation attacks the tonsil, it is influenced in its progress by those constitutional states that so markedly affect the natural history of disease. Hence, it is important to recognize the presence of syphilis, tuberculosis, rheumatism, etc., in the constitution of any patient we may be treating for a tonsillitis.

In children, these diseases may be latent, but, none the less, they have a potent influence over the course of the malady under consideration. Therefore, we should always make ourselves familiar with the natural history of the parents, and, if any of these diseases are found, so modify our treatment as to meet and counteract whatever of baleful influence may have been transmitted to the child.

In the suggestions to follow, on the management of an acute tonsillitis in children, it must be understood that no routine practice is proposed. The plan detailed must be so modified as to meet the hereditary and acquired variations from health in the particular case under consideration.

In order to obtain a clear idea of what is required in a rational treatment of a tonsillitis, let us see how an inflammation may behave when attacking that organ. In our opinion, there has been too much refinement in this matter. Bearing in mind its anatomical structure, we observe, in the first place, that an inflammation may limit itself entirely to the tissue immediately surrounding the tonsil, and then we have the peritonsillitis of some authors; it may express itself in the superficial parts, and become the erythematous tonsillitis of others; it may be deep-seated, involving the parenchyma, and we have the parenchymatous tonsillitis, or the true quinsy of the older writers; and again, the brunt of the inflammation may be confined to the

* Read before the Buffalo Obstetrical Society, March 27, 1888.

lacunæ, and then the disease is called folliculous tonsillitis. Now, in our opinion, this is the same inflammation, modified according to the constitutional state of the patient, the kind and severity of the exposure, and so on. As an illustration, it has been observed that the variety of tonsillitis called parenchymatous, occurs with great frequency in rheumatic subjects, and treatment followed in recognition of this fact—as the exhibition of the salicylates, salol, etc.,—has resulted in prompt relief. Other instances could be cited in proof of this position, but it would carry us too far from the immediate purpose of this paper. The question before us is, how to treat a case of simple tonsillitis, by which is meant, one uncomplicated by any other disease, and uninfluenced by the presence of any diathesis. Such cases are not rare, and, in our opinion, can be greatly modified in their duration and severity by proper treatment.

We have to deal with a sthenic inflammation—one that develops very rapidly, and continues at a great height for some days. The plain indication, then, is to control the production of this heat, to so influence the nerve centers as to make a high temperature impossible. This is done by the exhibition of antipyretics. So much for the general treatment. The next indication is to relieve the local distress. When the mucous membrane of the mouth and throat is inflamed, the secretion therefrom is highly acid. This acid secretion acts, in time, as an irritant, and keeps up the local disturbance. The indication is to apply alkalies to the surface of the tonsil, to neutralize the acidity of the secretions, and relieve the inflamed surface of this great source of irritation.

This is the general plan proposed; the details of its application are as follows:

The doses given are for adults, for the reason that we then have a definite standard to go by, which can be modified to meet the age of each individual case.

First, to keep down the temperature:

The various antipyretics may be used according to personal choice, but we have come to rely principally upon antifebrin.

This is to be given in five grain doses every hour until the temperature falls to nearly normal, and then at intervals necessary to prevent it rising again. We have never been obliged to give more than three doses in order to accomplish the first indication; generally two doses have been sufficient. In children, the minimum dose according to age should be given, and the patient carefully watched. Occasionally, it will be found to have a depressant effect, and must be abandoned for one of the other antipyretics.

The local treatment can be applied in several ways. Bicarbonate of sodium can be dusted upon the tonsils by means of an ordinary powder-blower, or a solution, ten grains to the ounce of water, can be sprayed on the parts by means of an atomizer, or, where the patient is of sufficient age, he can be instructed to dip the finger into the powder and touch the surface of the tonsil with it, or he can hold the solution in the mouth, allowing it to bathe the parts for a few moments. This local treatment should be used frequently, say at intervals of an hour, during the day.

Our notes show that, with this plan of treatment, four cases of severe tonsillitis, seen within the last few months, were limited to two days each. On the third day, there remained simply the general malaise, which is apt to follow cases of this kind. The temperature of these cases, when first seen by the writer, ranged from 102° to 104° F.

Professional friends, to whom this treatment was suggested, have reported equally good results. It is not necessary to report these cases in detail, but we content ourselves by formulating the conclusions of this paper as follows:

I. When an inflammation attacks the tonsil, it is greatly influenced in its course by the presence of any diathesis.

II. The treatment must be so arranged as to meet and counteract the influence of this diathesis.

III. In all cases, simple as well as complicated, the general indications are to keep down the temperature and to relieve the local irritation.

IV. The first indication can be met by the exhibition of antifebrin in proper doses; the second by the frequent application of bicarbonate of sodium, either in powder or in solution, to the surface of the tonsil.

V. This plan, properly followed, will generally limit the disease from one to three days.

273 Franklin street.

